# Different Regulation of Physiological and Tumor Angiogenesis in Zebrafish by Protein Kinase D1 (PKD1)

**DOI:** 10.1371/journal.pone.0068033

**Published:** 2013-07-09

**Authors:** Marcus Hollenbach, Sandra Jasmin Stoll, Kristina Jörgens, Thomas Seufferlein, Jens Kroll

**Affiliations:** 1 Department of Vascular Biology & Tumorangiogenesis, Center for Biomedicine and Medical Technology Mannheim, Medical Faculty Mannheim, Heidelberg University, Heidelberg, Germany; 2 Department of Internal Medicine I, Martin Luther University Halle-Wittenberg, Halle, Germany; 3 Division of Vascular Oncology and Metastasis, German Cancer Research Center (DKFZ-ZMBH Alliance), Heidelberg, Germany; 4 Department of Internal Medicine I, Ulm University, Ulm, Germany; University of Nebraska Medical Center, United States of America

## Abstract

Protein kinase D isoenzymes (PKDs, Prkds) are serine threonine kinases that belong to the CAMK superfamily. PKD1 is expressed in endothelial cells and is a major mediator of biological responses downstream of the VEGFRs that are relevant for angiogenesis such as endothelial cell migration, proliferation and tubulogenesis *in vitro*. PKDs also play a critical role in tumor development and progression, including tumor angiogenesis. However, given the plethora of signaling modules that drive angiogenesis, the precise role of PKD1 in both physiological and tumor angiogenesis *in vivo* has not been worked out so far. This study aimed at dissecting the contribution of PKD1 to physiological blood vessel formation, PKD1 was found to be widely expressed during zebrafish development. As far as physiological angiogenesis was concerned, morpholino-based silencing of PKD1 expression moderately reduced the formation of the intersomitic vessels and the dorsal longitudinal anastomotic vessel in *tg(fli1:EGFP)* zebrafish. In addition, silencing of PKD1 resulted in reduced formation of the parachordal lymphangioblasts that serves as a precursor for the developing thoracic duct. Interestingly, tumor angiogenesis was completely abolished in PKD1 morphants using the zebrafish/tumor xenograft angiogenesis assay. Our data in zebrafish demonstrate that PKD1 contributes to the regulation of physiological angiogenesis and lymphangiogenesis during zebrafish development and is essential for tumor angiogenesis.

## Introduction

The protein kinase D (PKD, Prkd) family of serine threonine kinases belongs to the calcium/calmodulin-dependent protein kinase superfamily [1] and comprises three isoforms [2]. All isoforms share the same molecular structure, consisting of a C-terminal kinase domain and a variable N-terminal regulatory domain with two highly conserved cysteine-rich zinc finger-like motifs (termed C1a and C1b) and a pleckstrin homology domain. The N-terminal domain, and the zinc finger motifs in particular, are important in regulating PKD subcellular localization [3]. PKDs are activated by various stimuli, including phorbol esters, G-protein-coupled receptors and reactive oxygen species [4]. PKDs act as prominent downstream targets of PKCs, including novel PKCη and PKCε [5], [6]. PKCs directly activate PKDs via phosphorylation at two critical serine residues within the activation loop of the catalytic domain [7]. However, PKDs can also be activated by direct binding of diacylglycerol (DAG) to the C1a domain [8].

PKD1 is expressed in a wide range of cells and tissues like fibroblasts, endothelial cells and dendritic cells and participates in a number of intracellular signaling pathways to regulate essential processes including cell survival, proliferation, cell motility, membrane trafficking, and immune response [9]–[13]. PKD1 is essential for normal embryogenesis. PKD2 is the major PKD isoform expressed in lymphoid tissues, but PKD2 catalytic activity is not essential for the development of mature peripheral T- and B-lymphocytes. PKD2 is a key feature in regulating the function of mature peripheral lymphocytes during adaptive immune responses [14].

Recent evidence indicates that PKD activation is required for biological responses to vascular endothelial growth factor (VEGF-A) in endothelial cells and important for crucial events in angiogenesis, such as migration, proliferation and tubulogenesis *in vitro* [10], [15]. PKDs are activated in endothelial cells upon stimulation by VEGF by two distinct mechanisms involving different tyrosine phosphorylation sites on the VEGF receptor 2 (VEGFR2). One pathway mediates VEGF-induced PKD activation and involves phosphorylation of Tyr951 in VEGFR2, which in turn leads to activation of PLCγ and generation of DAG and inositol-1,4,5 trisphosphate (IP3). DAG recruits both PKDs to the cell membrane where they are activated primarily by novel PKC isoforms. Alternatively, activation of the VEGFR2 induces tyrosine phosphorylation of PKDs, tyrosine phosphorylated PKD mediates VEGF-induced proliferation [16], [17].

Recent data from our laboratory identified PKD isoforms as novel, essential mediators of tumor cell - endothelial cell communication [18]: PKDs regulate both hypoxia-induced VEGF expression/secretion by the tumor cells and VEGF stimulated angiogenesis. PKDs therefore constitute potential therapeutic targets. Targeting PKDs may not only impinge on tumor cell survival and proliferation directly, but also indirectly by an inhibitory effect on tumor vascularization. Several selective PKD inhibitors are currently under investigation [16], [19]. However, angiogenesis is a complex process that is regulated by multiple signaling pathways. Furthermore, physiological angiogenesis and tumor angiogenesis share some common features, but do also exhibit distinct characteristics [20]. The precise contribution of PKDs to physiological angiogenesis compared to tumor angiogenesis *in vivo* is currently elusive.

The zebrafish *(Danio rerio)* is a small tropical freshwater fish that allows both the study of embryonic development including vasculogenesis and angiogenesis [21], and tumor angiogenesis *in vivo* in the same model. The model is particularly interesting since zebrafish share many genes and mechanisms of angiogenesis regulation with mammals [22]. Vasculogenesis in zebrafish is characterized by formation of the dorsal aorta and the posterior cardinal vein whereas angiogenesis comprises the formation of intersomitic vessels and the dorsal longitudinal anastomotic vessel. The formation of the intersomitic vessels and the subintestinal veins in early embryos can easily be monitored making zebrafish a suitable model for the study of angiogenesis inhibitors [23], [24]. Various cancer cell lines induce neovascularization when xenografted in the zebrafish [25]–[31].

PKD1 (XM_680127) is located on chromosome 17 in zebrafish, the transcript has a length of 3419 bp and 18 exons [32]. Here we aimed at dissecting the contribution of PKD1 to physiological angiogenesis and tumor angiogenesis *in vivo* in zebrafish. Our data show that PKD1 is widely expressed in zebrafish and contributes to physiological angiogenesis but is crucial only for tumor angiogenesis in the zebrafish/tumor xenograft angiogenesis assay. These data define a distinct role for PKD in physiological angiogenesis compared to tumor angiogenesis and suggest that in particular tumor angiogenesis is critically dependent on PKD making the kinase indeed an interesting target for tumor therapy.

## Material and methods

### Zebrafish Lines, Cell Lines, Antibodies and Reagents

Embryos of AB wild type, the *tg(fli1:EGFP)* and *tg(nflk:EGFP)* line [33] were raised and staged as described [34]. Embryos were kept in E3 solution at 28.5°C with or without 0.003% PTU (1-phenyl-2-thiourea) (Sigma) to suppress pigmentation and staged according to hours post fertilization (hpf). HCT116 human colon carcinoma cells were cultured in DMEM Glutamax (Gibco) supplemented with 10% heat-inactivated fetal calf serum and antibiotics.

We used the following antibodies for this study: rabbit anti-PKD1 (PKCµ C20, SC-639, Santa Cruz Biotechnology, epitope mapping at the c-terminus of PKD1 of mouse origin), rabbit anti-PKD1 148 (kind gift of Johan van Lint, Department of Molecular Cell Biology, University of Leuren, Belgium), goat anti-actin (I-19, SC-1616, Santa Cruz Biotechnology), rabbit anti-GFP (A-11122, Invitrogen), Alexa-546 coupled secondary antibodies (goat anti-rabbit, A11010, Invitrogen) and HRP-conjugated antibodies (goat anti-rabbit P0448 and rabbit anti-goat P0160, DAKO).

### Whole Mount Antibody Staining and Immunohistochemistry

For whole mount antibody stainings *tg(fli1:EGFP)* embryos were fixed 2 h in 4% PFA/PBS, washed, dehydrated in methanol and stored at −20°C. Embryos were rehydrated, permeabilized with proteinase K (Macherey-Nagel) and fixed again with 4% PFA/PBS. After blocking in 1% BSA plus 2% serum in PBST, embryos were incubated with an anti-GFP antibody or PKD1 antibody at 4°C overnight. On the following day embryos were washed for 6 h for anti GFP- and 90 minutes for anti PKD1-stainings. The secondary antibody was added at 4°C overnight for GFP- and two hours at room temperature for PKD1-stainings. The colour reaction was developed using the Vectastain ABC kit with horseradish peroxidase and DAB as a chromogen.

For immunostaining of zebrafish sections *tg(fli1:EGFP)* embryos were fixed overnight at 4°C in 4% PFA/PBS, washed, kept in 18% sucrose/PBS overnight at 4°C, embedded in Tissue-Tek (Sakura Finetek Europe) and stored at −80°C. 10 µm sections were made using a microtome (Leica CM-1900). Sections were washed, re-fixed, blocked in 3% BSA/PBS and incubated overnight at 4°C with the PKD1 antibody and subsequently with Alexa-546 coupled secondary antibody.

### Western Blot Analysis

Zebrafish embryos were washed with PBS and deyolked by pipetting up and down to destroy the yolk sac. Embryos were collected by centrifugation (5 min, 14.000 rpm, 4°C) and supernatants containing the yolk were discarded. Deyolked embryos were resuspended in lysis-buffer (150 mmol/L NaCl, 50 mmol/L Tris-HCl, pH 7.4, 1% NP40, 10 mmol/L EDTA, 10% glycerol, and protease inhibitors) followed by homogenization with a syringe and incubated for 30 min on ice. After centrifugation for 5 minutes supernatant was used for western blot analysis. The protein-lysates were boiled, and separated by SDS-PAGE, transferred to nitrocellulose membrane and incubated with the indicated antibodies, followed by incubation with Western blot detection reagent (Ace-Glow solution, 37–3420, Peqlab). Western blot signals were quantified using Gel-Pro Analyzer 6.0, INTAS and normalized to its respective loading controls.

### RT-PCR

Total RNA was isolated from zebrafish embryos using the RNeasy Mini-Kit (Qiagen) following the manufacturer’s protocol. First-strand cDNA was generated from normalized RNA amounts using random hexamer primers and the Superscript first strand kit (Invitrogen). RT-PCR was performed with pfu-polymerase (Fermentas) and specific primer pairs: zebrafish actin (413 bp fragment; forward: CTTGCGGTATCCACGAGAC, reverse: GCGCCATACAGAGCAGAA; Program: 95°C for 3 min, (95°C for 45 s, 56°C for 45 s, 72°C for 3 min)×30, 72°C for 7 min), zebrafish PKD1 (799 bp fragment; forward: AGAGGCCGTTCCAATTCCCAGTCC, reverse: CGTCGGCTCGGCTCAGGTTCTC), zebrafish PKD2 (421 bp fragment; forward: TTTGTCCAGTTTCCTGAATGC, reverse: GGCTACTAAGGGACTCGGTTG) and zebrafish PKD3 (423 bp fragment; forward: TGATTCAGAGGAACCCACAAC, reverse: TCCCCCACGTAGTACACCATA). For zebrafisch PKD1-, PKD2- and PKD3-primer pairs we used the following PCR program: 95°C for 3 min, (95°C for 45 s, 56°C for 45 s, 72°C for 3 min)×45, 72°C for 7 min. To analyze expression of PKD1 in EGFP positive cells (mainly endothelial cells and some pharyngeal arch and red blood cells) we used cDNA from purified cells using flow cytometry as recently described [35].

### Injections of mRNA and Morpholinos

For mRNA synthesis we used the previously generated PKD1-pCDNA3 vector (a kindly gift of Tim Eiseler, Institute of Molecular Cell Biology and Immunology, University of Stuttgart [36]) and the T7 message machine kit 1346 (Ambion) following the manufacturer’s protocol. RNA was diluted in 0,1 M KCl to concentrations of 100 ng/µl. Morpholinos were diluted in 0,1 M KCl to concentrations of 0.1–2 µg/µl. One nanoliter of each dilution was injected through the chorion of 1-cell or 2-cell stage embryos using borosilicate-needles and pneumatic picco pump (World Precision Instruments).

The following splice-blocking morpholinos (SB-Mo) (Gene Tools) were used: PKD1-SB-Mo I5∶5′-CAAATCTCCGTTTCTTGACACCTCT-3′ (targeting intron5-exon5 junction) PKD1-SB-Mo I6∶5′-GGCTGATGATCCTGTTGGACTCATC-3′ (targeting intron6-exon6 junction) Standard Control-Mo (Co-Mo).

### Injection of HCT116 Cells

HCT116 tumor cells were cultivated as described above. On day of injection tumor cells were detached by trypsin at a confluence of 70–80%. Cells were washed in PBS two times and resuspended in matrigel (Becton Dickinson, 354230) to a final concentration of 4×10^6^/30 µl. The suspension was loaded in precooled borosilicate-needles. Approximately 4–10 nl of the tumor/matrigel suspension was injected per embryo in the perivitelline space of 48 hpf dechorionated *tg(fli1:EGFP)* zebrafish embryos [30]. Injected embryos were incubated for additional 48 hours in E3 solution with 0.003% PTU (1-phenyl-2-thiourea) (Sigma). At 96 hpf embryos were anesthetized with 0.003% tricaine and analyzed using a fluorescence microscope. Labeling of HCT116 cells was performed before injection using the Vybrant® DiI Cell-Labeling Solution (V-22885, Invitrogen) reagent following the instructions of the manufacturer. HCT116 cells were purchased from ATCC®; Number: CCL-247™. HCT116 cells were validated to be authentic by DNA profiling using 8 different and highly polymorphic short tandem repeat (STR) loci and were further negatively tested for presence of mitochondrial DNA sequences from rodent cells as mouse, rat, chinese and syrian hamster by the German Biological Resource Centre DSMZ/Braunschweig/Germany.

### Angiogenesis Inhibitor Vatalanib (PTK787)

To study effect of the VEGF receptor inhibitor Vatalanib/PTK787 (Selleckchem) on physiological angiogenesis *tg(fli1:EGFP)* zebrafish embryos were incubated after fertilization for 48 hours in E3 solution containing 0.003% PTU and 0.1 µM Vatalanib. Vatalanib was dissolved in DMSO and control embryos were kept in E3 solution with 0.003% PTU and corresponding dilutions of DMSO. At 48 hpf embryos were analyzed by confocal microscopy. To analyze the effect of Vatalanib on tumor angiogenesis we injected HCT116 cells in 48 hpf embryos as described above. After injection embryos were incubated for additional 48 hours in E3 solution containing 0.003% PTU and 0.1 µM Vatalanib. At 96 hpf embryos were anesthetized with 0.003% tricaine and analyzed with fluorescence microscopy.

### Statistical Analysis and Quantification

Results are expressed as mean ± SD. Comparisons between groups were analyzed by Student’s t-test (two-sided). P values <0.05 were considered as statistically significant. For quantification of the vasculature in zebrafish, *tg(fli1:EGFP)* embryos were stained using an anti-GFP antibody with a DAB-based protocol and analyzed under the microscope. Embryos with vascular defects in the intersomitic vessels (ISVs) and DLAVs were counted and the percentage of embryos with defects per group was calculated. At least 30 embryos per group were counted. For each morpholino or RNA injection the first 17 ISVs from the anterior part of at least 30 embryos (48 hpf) were analyzed for ISV formation. For quantification of the dorsal longitudinal anastomotic vessel (DLAV), DLAV segments between 17 ISVs from the anterior part of at least 30 embryos (48 hpf) were counted. To analyze formation of the thoracic duct (TD) by confocal microscopy we counted at least 30 embryos for each group. Defects in the TD constitute either a discontinuous or complete missing TD. For quantification of tumor induced neoangiogenesis after injection of HCT116 cells the sprouting length of the subintestinal venous plexus (SIV) per embryo and the cumulative sprouting length of at least 30 embryos were analysed at 96 hpf. For cumulative sprouting-length all sprouts of the same number of embryos per group were summed.

### Microscopy and Analysis

For *in vivo* imaging, EGFP-expressing *tg(fli1:EGFP)* embryos were manually dechorionated, anesthetized with 0.003% tricaine and embedded in 1% low-temperature melting agarose (Roth). Fluorescence was analyzed using the confocal microscope DMIRE2 with Leica TCS SP2 True Confocal Scanner (Leica Microsystems). Stacks were recorded as indicated in the overview image. Overview pictures and sections of embryo as well as SIV-sprouting were analyzed with the fluorescence microscope DMI 6000 B (Leica).

## Results

### PKD1 is Widely Expressed in Zebrafish Embryos

**Figure 1:**
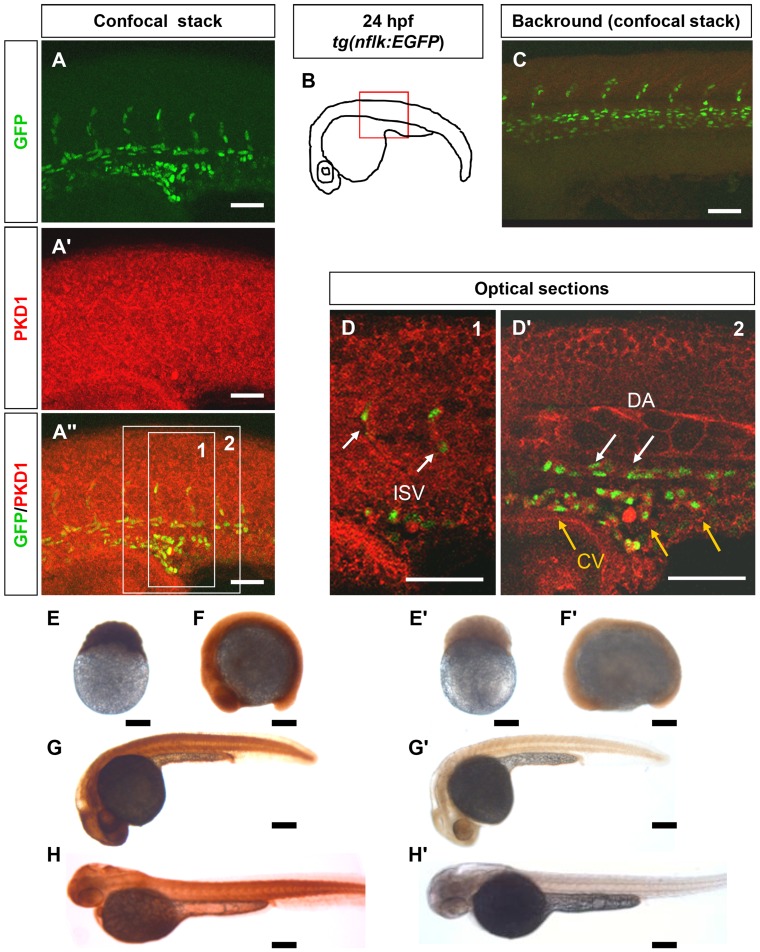
PKD1 expression in 24 hpf *tg(nflk:EGFP*) zebrafish embryo and in zebrafish development. A–A″, Whole mount antibody staining for PKD1 in 24 hpf *tg(nflk:EGFP*) zebrafish embryos. Pictures show whole confocal stacks of several optical sections. GFP signal is shown in green (A), PKD1 staining in red (A′), (A″) displays the merge. Box 1 and 2 mark the area where single optical sections were selected from (D, D′). B, Scheme of a 24 hpf embryo. The red box marks the area of confocal images in (A). C, for background control, embryo was stained without antibody against PKD1. D, Optical sections of the embryo, where colocalization of nuclear GFP signal in endothelial cells (green) and PKD1 expression (red) is shown. ISV: intersomitic vessel. DA: dorsal aorta. CV: cardinal vein. E–H′, Expression of PKD1 during zebrafish embryogenesis indicated ubiquitous distribution of the PKD1 protein at the 256-cell stage, 14-somite stage, 24 hpf and 48 hpf as shown by whole mount PKD1 antibody staining (E–H). E′–H′, Whole mount antibody control stainings lacking the primary PKD1 antibody. White scale bars: 100 µm; black scale bars: 300 µm.

To analyze expression of PKD1 in zebrafish we performed whole mount antibody staining of *tg(nflk:EGFP)* zebrafish embryos (Fig. 1, fig. S1). Confocal stacks of stained embryos displayed ubiquitous expression of PKD1 in 24 hpf (Fig. 1 A–A″) and 48 hpf (fig. S1 A–A″) zebrafish embryos. To confirm coexpression with endothelial cells, optical longitudinal sections are shown (Fig. 1 D–D′, fig. S1 D–D′). Green fluorescent nuclei of endothelial cells from the trunk vasculature - including the dorsal aorta (DA), the posterior cardinal vein (CV), the intersomitic vessels (ISV) and the dorsal longitudinal anastomotic vessel (DLAV) - colocalized with the cytoplasmatic signal of PKD1 antibody staining. Furthermore, whole mount PKD1 staining of several developmental stages of *tg(fli1:EGFP)* zebrafish embryos display ubiquitous PKD1 expression during early and late embryonic development starting from the 256 cell-stage until 48 hpf (Fig. 1 E–H′). At 48 hpf zebrafish embryo cross-sections showed PKD1 expression in all tissues examined (fig. S1 E–J). Merged images consisting of PKD1 antibody staining and EGFP fluorescence of *tg(fli1:EGFP)* zebrafish embryos revealed a high expression of PKD1 in the trunk vasculature similar to the longitudinal sections described above. Endothelial cell expression of PKD1 was also confirmed by RT-PCR from endothelial cells purified from *tg(fli1:EGFP)* embryos (fig. S2 E). Thus, PKD1 is widely expressed during zebrafish development and in the developing vascular system.

### PKD1 Regulates Physiological Angiogenesis

**Figure 2:**
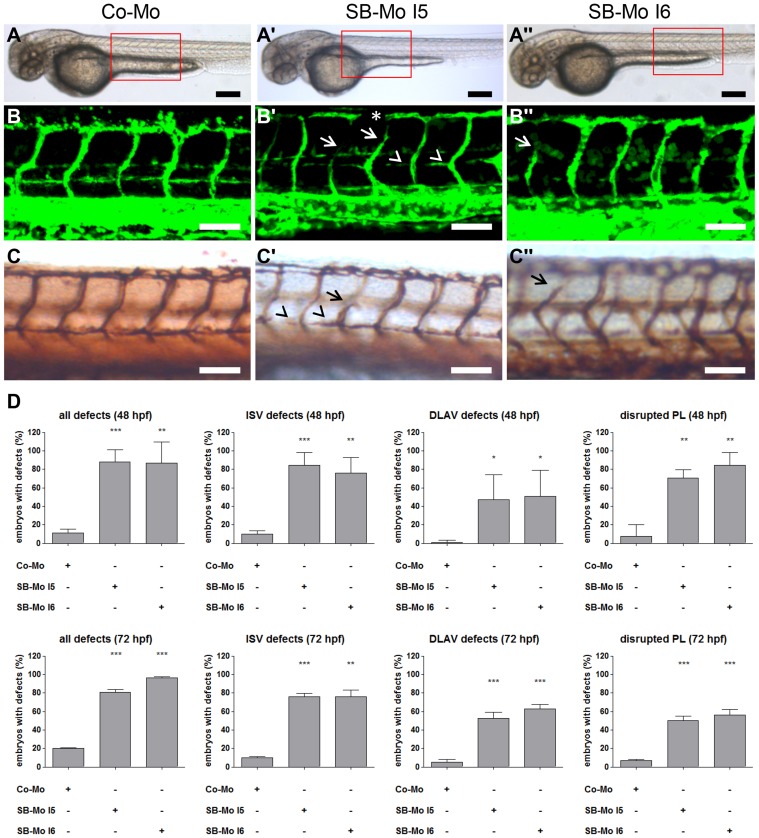
PKD1 expression silencing resulted in slight vascular defects in *tg(fli1:EGFP)* zebrafish embryos. A–A″, Overall morphology of Co-Mo - injected (2 ng) or PKD1 SB-Mo I5 or I6 - injected (500 pg) 48 hpf embryos did not show morphological alterations. Red boxes indicate regions of pictures shown in (B–C″). B–C″, Embryos were analyzed at 48 hpf by confocal microscopy (B–B″) or anti-GFP antibody staining (C–C″). Morpholino-based silencing of PKD1 in zebrafish disrupted partially formation of intersomitic vessels (ISVs) (arrows), the dorsal longitudinal anastomotic vessel (DLAV) (asterisks), and the parachordal lymphangioblasts (PL) (arrowheads) (B–C″). D, Quantification of the number of embryos with vascular defects, ISV defects, DLAV defects and disrupted PL at 48 hpf and 72 hpf. For each group at least 40 embryos were analyzed. White scale bars: 100 µm; black scale bars: 300 µm. *P<0.05, **P<0.01, ***P<0.001.

**Figure 3:**
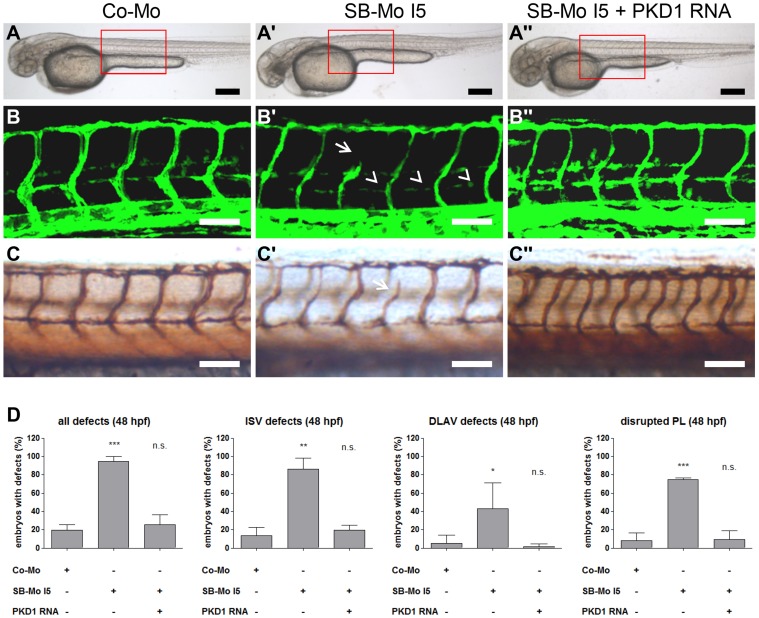
Injection of human PKD1 mRNA rescued vascular defects in PKD1 morphant *tg(fli1:EGFP)* zebrafish embryos. A–A″, Overall morphology of Co-Mo - injected (2 ng), PKD1 SB-Mo I5 - injected (500 pg) or of SB-Mo I5 and PKD1 mRNA (100 pg) co-injected fish embryos showed no morphological alterations. Red boxes indicate regions of pictures shown in (B–C″). B–C″, Embryos were analyzed at 48 hpf by confocal microscopy (B–B″) or anti-GFP antibody staining (C–C″). Morpholino-based expression silencing of PKD1 in zebrafish disrupted partially formation of the vasculature (ISVs (arrows), PL (arrowheads)) (B′, C′). Coinjection of SB-Mo I5 (500 pg) and PKD1 mRNA (100 pg) rescued the vascular malformation in PKD1 morphants (B″, C″). D, Quantification of the number of embryos with vascular defects, ISV defects, DLAV defects and disrupted PL at 48 hpf. For each group at least 40 embryos were analyzed. Black scale bars: 300 µm; white scale bars: 100 µm. *P<0.05, **P<0.01, ***P<0.001.

Next we examined the contribution of PKD1 to physiological angiogenesis *in vivo* by silencing of the kinase. We analyzed formation of the vasculature in *tg(fli1:EGFP)* zebrafish embryos in the presence of two PKD1 specific splice-blocking morpholinos (SB-Mo I5 and I6, Fig. 2) and human PKD1 mRNA (Fig. 3). Injection of SB-Mo I5 or SB-Mo I6 efficiently reduced PKD1 mRNA and protein levels at 24 hpf (fig. S2 A, A′, B, B′, D and D′). As expected, injection of human PKD1 mRNA increased human PKD1 protein expression in the zebrafish embryos (fig. S2 C and C′). Silencing of PKD1 did not result in major morphological alterations. However, more than 80% of the PKD1 morpholino injected zebrafish embryos exhibited defects in the trunk vasculature (ISVs, DLAV and PLs). At 48 hpf some ISVs did not form correctly and the dorsal longitudinal anastomotic vessel (DLAV) was partially interrupted (Fig. 2 A–C″). Quantification at 48 hpf revealed approximately 80% embryos with ISV-defects, 50% embryos with DLAV-defects and 70% embryos with PL-defects for both morpholinos as compared to controls showing no vascular defects (p<0.01, Fig. 2 D, upper row). These defects were still observed at 72 hpf, indicating that they are not based upon a developmental delay due to morpholino injection (Fig. 2 D, lower row). Thus, PKD1 silencing leads to reproducible albeit moderate vascular defects in zebrafish. To address the question, whether loss of PKD1 is partially compensated by PKD2 or PKD3 expression, RT-PCR analysis with PKD1 morphants was performed (fig. S4). PKD1 silencing slightly increased PKD2 and PKD3 expression suggesting a compensatory upregulation of both kinases in PKD1 morphants.

To confirm that the observed vascular phenotype in the PKD1 morphants was specifically caused by silencing of PKD1 expression, human PKD1 sense RNA and PKD1 morpholino SB-Mo I5 were injected together. Coinjection of human PKD1 mRNA and a PKD1 morpholino completely rescued the vascular phenotype described above in the PKD1 morphants as demonstrated by confocal images (Fig. 3 B–B″), whole mount antibody staining (Fig. 3 C–C″) and quantification of the defects observed in zebrafish embryos (Fig. 3 D). Injection of PKD1-mRNA alone did not induce alterations in the developing vasculature of zebrafish embryos (data not shown). Next, we examined a potential role of PKD1 in cranial angiogenesis in zebrafish at 24 hpf and 48 hpf. Interestingly, silencing of PKD1 did not reveal vascular defects in the primordial hindbrain channel or central arteries (fig. S3) suggesting different regulatory mechanisms in the cranial and trunk vasculature in zebrafish. Taken together our findings suggest that PKD1 moderately contributes to physiological angiogenesis, particularly ISV and DLAV formation in zebrafish.

### PKD1 is Essential for Tumor Angiogenesis in Zebrafish

PKDs regulate proliferation, cell survival, migration and invasion [19]. Recently, we could implicate PKDs in tumor angiogenesis in the chicken CAM and in an orthotopic mouse model [18].

**Figure 4:**
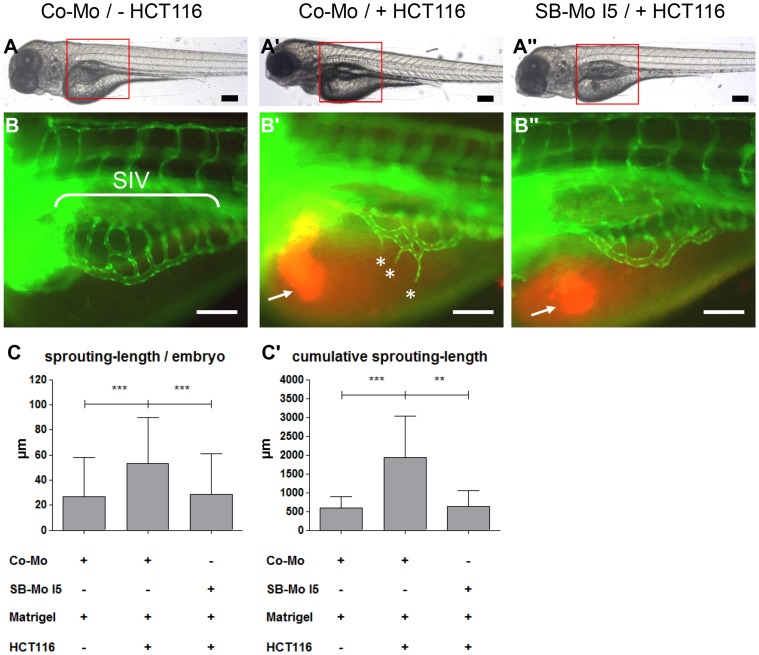
Tumor angiogenesis is abolished in PKD1 morphant *tg(fli1:EGFP)* zebrafish embryos. A–A″, Overall morphology of Co-Mo - injected (2 ng) or PKD1 SB-Mo I5 - injected (500 pg) 96 hpf embryos. At 48 hpf 1–4 nl of Matrigel (A, B) or Matrigel/HCT116 solution (A′, A″, B′, B″) was injected in the perivitelline space. Red boxes indicate regions of pictures shown in (B–B″). B–B″, HCT116 induced tumor angiogenesis as indicated by sprouting of subintestinal venous plexus (SIV) was analyzed at 96 hpf in *tg(fli1:EGFP)* embryos. Injection of HCT116 tumor cells (labeled with VybrantDil in red, arrow) led to a strong formation of ectopic blood vessels originated from the SIV (asterisks). In PKD1 morphants ectopic blood vessel formation was completely blocked. C–C′, Quantification of sprouting length per embryo (C) and cumulative sprouting length (C′) with S.D. of at least 30 embryos per group. For cumulative sprouting-length all sprouts of the same number of embryos per group were summed, (C′) represents means of three independent experiments with S.D. Black scale bars: 300 µm; white scale bars: 100 µm. *P<0.05, **P<0.01, ***P<0.001.

To analyze the function of PKD1 in tumor angiogenesis in zebrafish we used the zebrafish/tumor xenograft angiogenesis assay [30]. Co-Mo or PKD1 SB-Mo I5 were injected in the 1-cell or 2-cell stage of *tg(fli1:EGFP)* zebrafish embryos followed by injection of a HCT116 tumor cell-matrigel solution in the perivitelline space at 48 hpf. At 96 hpf formation of ectopic vessels originating from the subintestinal venous plexus (SIV) was analyzed (Fig. 4). Injection of the HCT116 cells into the perivitelline space of a Co-Mo injected zebrafish embryo led to an increase of ectopic sprouts causing a duplication of the SIV-sprouting length per embryo compared to the Co-Mo injected embryos, which were injected with matrigel alone. The cumulative sprouting length was increased by 3-fold compared to the injection of a matrigel solution without HCT116 cells (Fig. 4 A, A′, B, B′, C, C′). In marked contrast, there was no ectopic sprouting of the SIV upon injection of the HCT116 tumor cell-matrigel solution into PKD1 morphant (SB-Mo I5 injected) zebrafish embryos (Fig. 4 A″, B″, C, C′). PKD1 expression silencing by SB-Mo I5 injection led to nearly the same SIV-spouting-length per embryo and cumulative sprouting-length as observed in control embryos in the absence of HCT116 cells (Fig. 4 C–C′). To further support these findings complementary mRNA based PKD1 gain-of-function experiments using the zebrafish/tumor xenograft angiogenesis assay were performed. Injection of PKD1 mRNA into the 1- or 2-cell stage followed by HCT116 tumor cell injection at 48 hpf led to an increase in ectopic sprouts originating from the SIV at 96 hpf (fig. S5).

**Figure 5:**
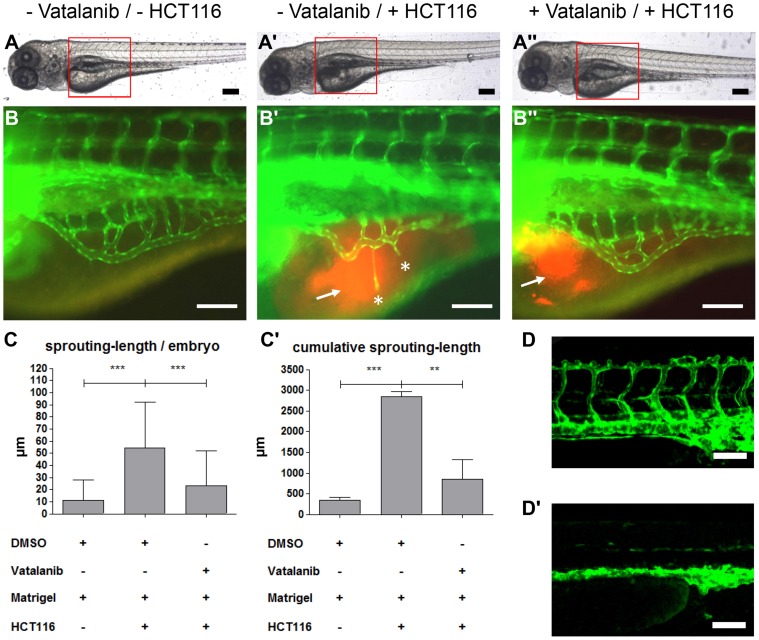
VEGF receptor-2 antagonist Vatalanib inhibited HCT116 induced tumor angiogenesis in *tg(fli1:EGFP)* zebrafish embryos. A–A″, Overall morphology of 96 hpf embryos after injection of 1–4 nl of Matrigel (A, B) or Matrigel/HCT116 solution (A′, A″, B′, B″) in the perivitelline space at 48 hpf. Directly after HCT116 tumor cell injection embryos were incubated in presence of 0.1 µM Vatalanib (A″, B″) or DMSO (A, A′, B, B′) for additional 48 hours. Red boxes indicate regions of pictures shown in (B–B″). B–B″, HCT116 induced angiogenesis as indicated by ectopic vessel formation originated from the subintestinal venous plexus (SIV) was analyzed at 96 hpf in *tg(fli1:EGFP)* embryos. Injection of HCT116 tumor cells led to strong ectopic vessel formation (asterisks) that was completely inhibited in Vatalanib treated zebrafish embryos. C–C′, Quantifications with S.D. of at least 30 embryos per group. For cumulative sprouting-length all sprouts of the same number of embryos per group were summed, (C′) represents means of three independent experiments with S.D. D–D′, Effect of Vatalanib on physiological angiogenesis in 48 hpf *tg(fli1:EGFP)* embryos. Embryos were incubated with DMSO (D) or 0.1 µM Vatalanib (D′) immediately after fertilization. Incubation with Vatalanib completely inhibited formation of ISV, DLAV and PL. Black scale bars: 300 µm; white scale bars: 100 µm. *P<0.05, **P<0.01, ***P<0.001.

The inhibitory effect of PKD1 silencing on the formation of ectopic sprouts originating from the SIV was compared to the effect of a pharmacological inhibitor of the VEGFR tyrosine kinase activity, Vatalanib (PTK787) [37]. Zebrafish embryos were incubated with Vatalanib starting at 48 hpf after HCT116-matrigel solution injection until observation at 96 hpf. Vatalanib treated zebrafish embryos showed a complete inhibition of ectopic sprout formation at 96 hpf that was comparable to the findings in the PKD1 zebrafish morphants (Fig. 5). To exclude effects of Vatalanib on physiological formation of subintestinal venous plexus we used a rather low inhibitor concentration (100 nM) causing no alterations in physiological angiogenesis or morphology (fig. S6). In conclusion, our data show that PKD1 is sufficient and also required for tumor angiogenesis of HCT116 in the zebrafish model demonstrating a major role of host-PKD1 in the regulation of tumor angiogenesis.

### Role of PKD1 in Zebrafish Lymphangiogenesis

**Figure 6:**
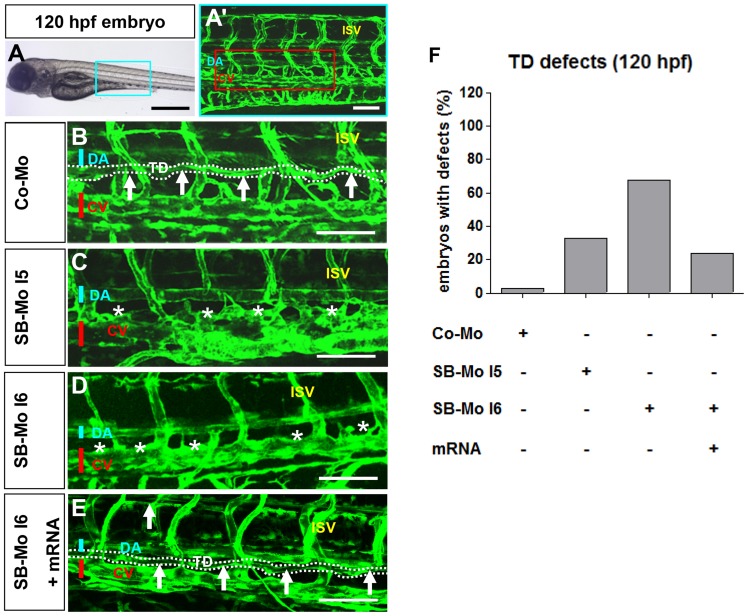
PKD1 expression silencing disturbed formation of thoracic duct in 120 hpf *tg(fli1:EGFP)* zebrafish embryos. A–A′, Bright-field image (A) and confocal microscopy (A′) of 120 hpf Co-Mo - injected (2 ng) zebrafish embryo showed physiological formation of thoracic duct (TD). The blue box marks the region shown in (A′). The red box marks the region shown in (B–E). DA: dorsal aorta, CV: cardinal vein, ISV: intersomitic vessel, TD: thoracic duct. C–D, Confocal microscopy of 120 hpf SB-Mo I5 - injected (500 pg) (C) and SB-Mo I6 - injected (500 pg) (D) embryos indicated a complete absence of TD (asterisks). E, Confocal microscopy of 120 hpf coinjected (500 pg SB-Mo I6 and 100 pg PKD1 mRNA) embryos showed a rescue effect on TD formation. F, Quantification of embryos with defects injected with Co-Mo (2 ng), SB-Mo I5 (500 pg), SB-Mo I6 (500 pg) or coinjection (SB-Mo I6 and mRNA). At least 30 embryos per group were analyzed. Blue bar indicates dorsal aorta (DA), red bar cardinal vein (CV) and arrows and dotted line the TD. Scale bars: 100 µm.

Apart from physiological angiogenesis and tumor angiogenesis lymphangiogenesis can also be examined in the zebrafish model. Here, early lymphangiogenesis is indicated by the appearance of the parachordal lymphangioblasts (PL). As it was shown in Fig. 2 and Fig. 3, PKD1 silencing inhibited PL formation. Since the PLs are the origin of the future lymphatic system [23], this implicates that PKDs can also contribute to the formation of the lymphatic system. Indeed, analysis of embryos at 120 hpf revealed the absence of the thoracic duct (TD) in about 30–70% of PKD morphants (Fig. 6 C, D, F) as compared to the control (Fig. 6 A–B). Conversely, coinjection of human PKD1 mRNA and PKD1 morpholino rescued the alterations in the developing lymphatic system represented by the complete formation of PLs at 48 hpf (Fig. 3) as well as defects in the TD at 120 hpf (Fig. 6 E, F). This data demonstrates that aside from physiological angiogenesis also lymphangiogenesis is influenced by PKD1.

## Discussion

PKD1 belongs to the calcium−/calmodulin-dependent protein kinase superfamily [1] and is expressed in a wide range of cells, including endothelial cells. PKD1 activity is required for the biological responses downstream of the VEGFR tyrosine kinase in endothelial cells and important for migration, proliferation and tubulogenesis, the relevant biological processes leading to angiogenesis [10], [15], [38]. PKD1 also regulates cell survival, cell motility, membrane trafficking, and immune response [9]–[13]. Furthermore PKD1 knockout mice revealed its essential role for normal embryogenesis and PKD2 is a key feature in regulating the function of mature peripheral lymphocytes during adaptive immune responses [14].

The aim of our study was to define the contribution of PKD1 to physiological and tumor angiogenesis in an established model system *in vivo*. The zebrafish model seemed to be appropriate for our purpose since it allows studying physiological angiogenesis and tumor angiogenesis *in vivo*. In addition, we found PKD1 to be widely expressed in early and late stages of embryonic zebrafish development. In particular, there was a marked PKD1 expression in the vascular system as revealed by optical longitudinal sections of whole mount stainings for PKD1. PKD1 signal was consistently colocalized in endothelial cells of the trunk vasculature in transgenic embryos with nuclear *flk1*:EGFP signal. Furthermore immunostaining of cross-sections and RT-PCR of sorted EGFP-positive endothelial cells supported this finding. Our data obtained by PKD1 silencing revealed that the kinase contributes to physiological angiogenesis in zebrafish: PKD1 depleted zebrafish exhibited defects in the intersomitic vessels and the dorsal longitudinal anastomotic vessel. Compared to other regulators of angiogenesis in zebrafish such as the ELMO1/DOCK180 complex [39] or the transcription factor HOXC9 [35] we detected a moderate phenotype but more than 80% of the PKD1 knockout embryos exhibited vascular defects. The moderate effect may result from a partial compensation of the loss of PKD1 by increased expression of PKD2 and PKD3. Additional, yet unknown pathways may also compensate the loss of PKD1. However, the effect of PKD1 on physiological angiogenesis was specific: All vascular defects caused by PKD1 silencing could be rescued by injecting human PKD1 sense RNA. This also demonstrates a highly conserved role and structure of PKD1. Taken together these findings demonstrate that PKD1 contributes to physiological angiogenesis downstream of the VEGFR2 in zebrafish.

Interestingly, zebrafish embryos lacking PKD1 exhibited also defects in the parachordal lymphangioblasts and in the thoracic duct. This indicates that PKD1 can also contribute to the development of the lymphatic system. This is the first time that PKDs have been implicated in lymphangiogenesis. Since zebrafish possess a lymphatic vascular system that shares the morphological, molecular and functional characteristics of the lymphatic vessels found in other vertebrates [40], PKD1 might play a role in lymphatic tumor metastasis. However, this issue requires further investigations.

Our data show that silencing of PKD1 almost completely prevented tumor angiogenesis induced by HCT116 cells [41] as determined by ectopic sprouting of the subintestinal venous plexus. Here the degree of inhibition by PKD1 silencing was comparable to the effect of the VEGFR tyrosine kinase-inhibitor Vatalanib. Former data demonstrated that PKDs regulate tumor cell-endothelial cell communication via the regulation of hypoxia-induced VEGF-A expression and VEGF-A stimulated blood vessel formation in pancreatic cancer [18]. Accordingly, our data strengthen the importance of PKDs in tumor angiogenesis. It would be therefore essential to investigate the role of PKD1 in other tumor types.

In a conclusion, PKD1 contributes to physiological angiogenesis and lymphangiogenesis *in vivo* and is critically required for tumor angiogenesis in the zebrafish model. Thus, PKD1 appears to play a distinct role in tumor angiogenesis compared to physiological angiogenesis. Tumor angiogenesis - at least in zebrafish - appears to be addicted to PKD signaling. As compared to established inhibitors of tumor angiogenesis like Bevacizumab, PKD1 represents a more specific therapeutic target anticipating less side effects than generally blocking VEGF since physiological angiogenesis is only slightly impaired by silencing of PKD1.

## Supporting Information

Figure S1 **PKD1 expression in 48 hpf zebrafish embryo.** A–A″, Whole mount antibody staining for PKD1 was performed in 48 hpf *tg(nflk:EGFP*) zebrafish embryos. Pictures show whole confocal stacks of several optical sections. GFP signal is shown in green (A), PKD1 staining in red (A′), (A″) displays the merge. Box 1 and 2 mark the area where single optical sections were selected from (D–D′). B, Scheme of a 48 hpf embryo. The red box marks the area of confocal images in (A). C, For background control, embryo was stained without antibody against PKD1. D, Optical sections of the embryo, where colocalization of nuclear GFP signal in endothelial cells and PKD1 expression is shown. E–J, Cross-sections of a 48 hpf *tg(fli1:EGFP)* embryo. E, Ubiquitous PKD1 expression (red). F, EGFP expression in the vasculature of a *tg(fli1:EGFP)* embryo, e.g. in the dorsal aorta (DA), posterior cardinal vein (PCV) and dorsal longitudinal anastomotic vessel (DLAV). G, Merge of (E) and (F). H, Confocal image of a 48 hpf *tg(fli1:EGFP)* zebrafish embryo confirmed co-localization of EGFP and PKD1 in the PCV, DA and DLAV. I, Bright field image. J, Control section lacking the primary PKD1 antibody revealed no staining. Scale bars: 100 µm (A–D′), 25 µm (E–J). ISV: intersomitic vessel. DLAV: dorsal longitudinal anastomotic vessel. PCV: posterior cardinal vein.
(JPG)

Figure S2 **PKD1 expression silencing by two splice-blocking morpholinos and PKD1 mRNA injection in zebrafish embryos.** A–A′, Expression silencing of PKD1 in 24 hpf zebrafish embryos using two splice-blocking morpholinos (SB-Mo I5 and I6). RT-PCR analysis of 2 ng control morpholino (Co-Mo) and 500 pg SB-Mo I5 (A) or 500 pg SB-Mo I6 (A′) injected embryos. The upper signal represents the wild-type (wt), the lower signal the morphant (mo) product. Injection of SB-Mo I5 or I6 generated a substantial loss of the wild-type splice product indicating the functionality of both PKD1 morpholinos. B–B′, PKD1 antibody stainings of cross-sections of 48 hpf embryos injected with CoMo (2 ng) (B) or SB-Mo I5 (500 pg) (B′) indicated reduced PKD1 expression (red colour) in PKD1 morphant embryos. Exposure time was 840 ms for all images. C, Injection of 100 pg human PKD1 sense RNA into zebrafish embryos led to an enhanced expression of PKD1 protein. C′, Quantification of (C) by densitometry, showing three independent experiments with S.D. D, Western blot analysis of 24 hpf embryos after injection of SB-Mo I5 (500 pg) or SB-Mo I6 (500 pg) indicated strong reduction in PKD1 protein expression as compared to Co-Mo - injected (2 ng) embryos. D′, Quantification of (D) by densitometry, results represent means of three independent experiments with S.D. E, Expression of PKD1 in *tg(fli1:EGFP)* zebrafish endothelial cells at 24 hpf as shown by RT-PCR of EGFP purified endothelial cells. Scale bars: 25 µm.
(JPG)

Figure S3 **PKD1 silencing in zebrafish did not alter cranial angiogenesis.** A–C′, 24 hpf (A, B, C) and 48 hpf (A′, B′, C′) *tg(fli1:EGFP)* zebrafish embryos were analyzed for defects in the primordial hindbrain channel (PHBC, arrows) and central arteries (CA, asterisks) by confocal microscopy. Injection of 500 pg SB-Mo I5 (B, B′) or 500 pg SB-Mo I6 (C, C′) did not reveal vascular defects in the cranial vasculature as compared to 2 ng Co-Mo injected *tg(fli1:EGFP)* zebrafish embryos (A, A′). Black scale bars: 500 µm, red scale bars: 50 µm
(JPG)

Figure S4 **Expression of PKD2 and PKD3 in PKD1 morphants.** A, RT-PCR expression analysis for PKD2 and PKD3 after PKD1 silencing in zebrafish using SB-Mo I5. Injection of SB-Mo I5 (500 pg) led to a weak increase of PKD2 and PKD3 expression at 48 hpf, 72 hpf and 96 hpf. B, Quantification of selected timepoints of (A) by densitometry; data represent means of three independent experiments with S.D. *P<0.05, **P<0.01, ***P<0.001.
(JPG)

Figure S5 **Increased tumor angiogenesis in** ***tg(fli1:EGFP)*** **zebrafish embryos overexpressing PKD1.** Overall morphology of orange-mRNA - injected (100 pg) or PKD1 mRNA - injected (100 pg) 96 hpf embryos. At 48 hpf 1–4 nl of Matrigel **(A, B)** or Matrigel/HCT116 solution **(A**′**, A″, B**′**, B″)** was injected in the perivitelline space. Red boxes indicate regions of pictures shown in **(B–B″)**. **B–B″**, HCT116 induced tumor angiogenesis as indicated by sprouting of subintestinal venous plexus (SIV) was analyzed at 96 hpf in *tg(fli1:EGFP)* embryos. Injection of HCT116 tumor cells (labeled with VybrantDil in red, arrow) led to a strong formation of ectopic blood vessels originated from the SIV (asterisks). In PKD1 overexpressing embryos ectopic blood vessel formation was further enhanced. **C–C**′, Quantification of sprouting length per embryo **(C)** and cumulative sprouting length **(C**′**)** with S.D. of at least 30 embryos per group. For cumulative sprouting-length all sprouts of the same number of embryos per group were summed, **(C**′**)** represents means of three independent experiments with S.D. Black scale bars: 300 µm; white scale bars: 100 µm. *P<0.05, **P<0.01, ***P<0.001.
(JPG)

Figure S6 **Effect of Vatalanib treatment on vascular development of the trunk vasculature in zebrafish embryos and overall morphology.** A–F′, *tg(fli1:EGFP) z*ebrafish embryos were treated with Vatalanib in different concentrations for 48 hours, beginning at 48 hpf. Confocal images of trunk vasculature (A–F) and light images of overall morphology (A′–F′) were taken at 96 hpf. Embryos were incubated with control solution (0.05% DMSO in eggwater equal to the DMSO concentration in 10 µM Vatalanib treated embryos) (A, A′), 0,1 µM Vatalanib (B, B′), 1 µM Vatalanib (C, C′), 2 µM Vatalanib (D, D′), 5 µM Vatalanib (E, E′) and 10 µM Vatalanib (F, F′). ISV = intersomitic vessel, SIV = subintestinal vein plexus, PL = parachordal lymphangioblasts, DLAV = dorsal longitudinal anastomotic vessel. White scale bar: 100 µm; black scale bar: 500 µm.
(JPG)
