# Correction: Different Regulation of Physiological and Tumor Angiogenesis in Zebrafish by Protein Kinase D1 (PKD1)

**DOI:** 10.1371/journal.pone.0231153

**Published:** 2020-03-25

**Authors:** Marcus Hollenbach, Sandra Jasmin Stoll, Kristina Jörgens, Thomas Seufferlein, Jens Kroll

After publication of this article [1], concerns were raised regarding the PKD1-targeting morpholino oligo (MO) described in the Methods. Specifically:

- The PKD1-SB-Mo I5∶5′-CAAATCTCCGTTTCTTGACACCTCT-3′ does not target intron5-exon5 junction and instead targets the exon7-exon8 sequence
- The PKD1-SB-Mo I6∶5′-GGCTGATGATCCTGTTGGACTCATC-3′ does not target intron6-exon6 junction and instead targets the exon8-exon9 sequences

The authors agree that the information given in the article regarding exon number in the prkd1 gene where the morpholinos bind is not correct. The authors provide the following clarification in response to the concerns:

The morpholinos were selected based on sequence XM_680127 during the time the study was performed. We note that record has since been removed “as a result of standard genome annotation processing” (https://www.ncbi.nlm.nih.gov/nuccore/XM_680127.5?report=genbank). An NCBI blast search for the two morpholinos used in this work found that both morpholinos are a 100% match for the gene XM_0051588.16.4 or XM_021467430.1 (Danio rerio protein kinase D1 (prkd1), transcript variants X1 and X2). According to the new sequences, morpholino PKD1-SB-Mo I5 binds in Exon 7 and Exon 8 (Exon 7: AGAGGTGTCAAGAAACGGAGA; Exon 8: TTTG) and morpholino PKD1-SB-Mo I6 binds in Exon 8 and Exon 9 (Exon 8: GATGAGTCCAACAGGATCATCAG; Exon 9: CC).
As shown in Supporting Information Figure S2 A and A’, injection of morpholino PKD1-SB-Mo I5 and PKD1-SB-Mo I6 strongly reduced the upper wildtype signal and generated new morphant signals migrating below the wildtype signal. In addition, our western blot analysis has proven a loss of prkd1 expression for zebrafish injected with both morpholinos (Figure S2 D). Based on this information, we have concluded that both morpholinos work very well and can be used for further studies. This correction does not change the interpretation of the data or the conclusions of the article.
