# Supplementary figures and images for: Different Regulation of Physiological and Tumor Angiogenesis in Zebrafish by Protein Kinase D1 (PKD1)
Source: PLoS One. 2013 Jul 9;8(7):e68033. doi: 10.1371/journal.pone.0068033 (PMC3706615; doi:10.1371/journal.pone.0068033)

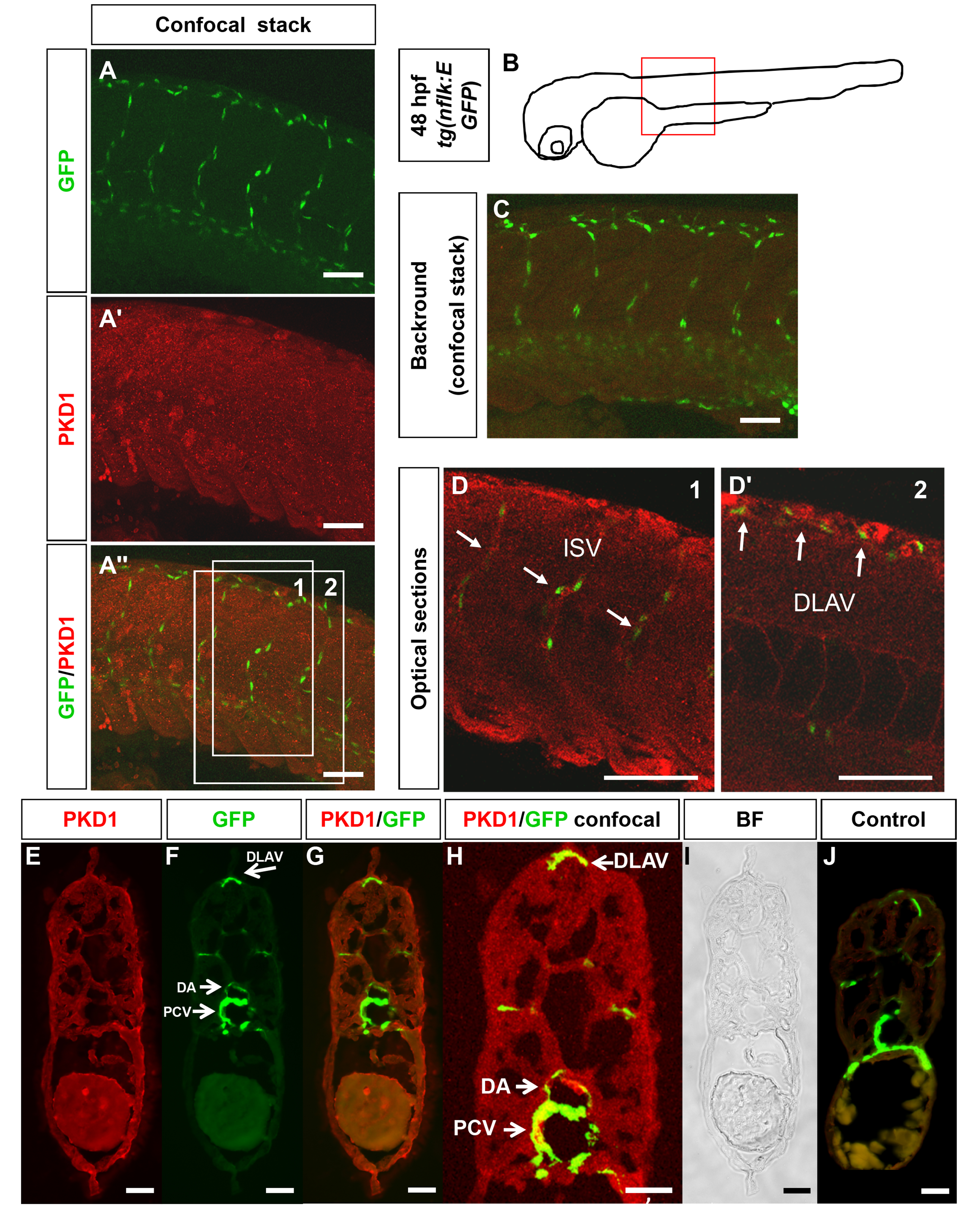

Supplement: Figure S1 — PKD1 expression in 48 hpf zebrafish embryo. A–A″, Whole mount antibody staining for PKD1 was performed in 48 hpf tg(nflk:EGFP) zebrafish embryos. Pictures show whole confocal stacks of several optical sections. GFP signal is shown in green (A), PKD1 staining in red (A′), (A″) displays the merge. Box 1 and 2 mark the area where single optical sections were selected from (D–D′). B, Scheme of a 48 hpf embryo. The red box marks the area of confocal images in (A). C, For background control, embryo was stained without antibody against PKD1. D, Optical sections of the embryo, where colocalization of nuclear GFP signal in endothelial cells and PKD1 expression is shown. E–J, Cross-sections of a 48 hpf tg(fli1:EGFP) embryo. E, Ubiquitous PKD1 expression (red). F, EGFP expression in the vasculature of a tg(fli1:EGFP) embryo, e.g. in the dorsal aorta (DA), posterior cardinal vein (PCV) and dorsal longitudinal anastomotic vessel (DLAV). G, Merge of (E) and (F). H, Confocal image of a 48 hpf tg(fli1:EGFP) zebrafish embryo confirmed co-localization of EGFP and PKD1 in the PCV, DA and DLAV. I, Bright field image. J, Control section lacking the primary PKD1 antibody revealed no staining. Scale bars: 100 µm (A–D′), 25 µm (E–J). ISV: intersomitic vessel. DLAV: dorsal longitudinal anastomotic vessel. PCV: posterior cardinal vein. (JPG) [file pone.0068033.s001.jpg]

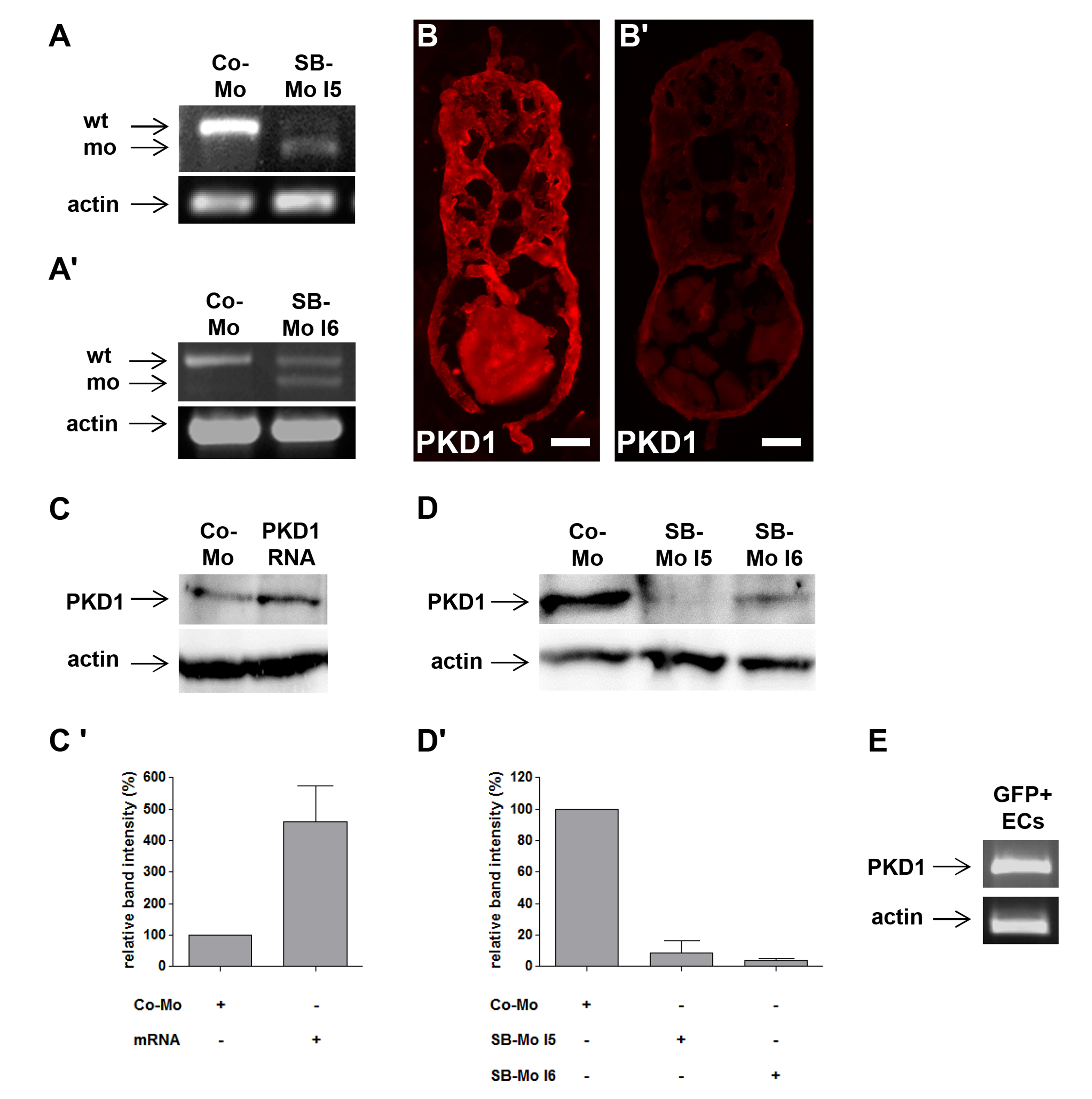

Supplement: Figure S2 — PKD1 expression silencing by two splice-blocking morpholinos and PKD1 mRNA injection in zebrafish embryos. A–A′, Expression silencing of PKD1 in 24 hpf zebrafish embryos using two splice-blocking morpholinos (SB-Mo I5 and I6). RT-PCR analysis of 2 ng control morpholino (Co-Mo) and 500 pg SB-Mo I5 (A) or 500 pg SB-Mo I6 (A′) injected embryos. The upper signal represents the wild-type (wt), the lower signal the morphant (mo) product. Injection of SB-Mo I5 or I6 generated a substantial loss of the wild-type splice product indicating the functionality of both PKD1 morpholinos. B–B′, PKD1 antibody stainings of cross-sections of 48 hpf embryos injected with CoMo (2 ng) (B) or SB-Mo I5 (500 pg) (B′) indicated reduced PKD1 expression (red colour) in PKD1 morphant embryos. Exposure time was 840 ms for all images. C, Injection of 100 pg human PKD1 sense RNA into zebrafish embryos led to an enhanced expression of PKD1 protein. C′, Quantification of (C) by densitometry, showing three independent experiments with S.D. D, Western blot analysis of 24 hpf embryos after injection of SB-Mo I5 (500 pg) or SB-Mo I6 (500 pg) indicated strong reduction in PKD1 protein expression as compared to Co-Mo - injected (2 ng) embryos. D′, Quantification of (D) by densitometry, results represent means of three independent experiments with S.D. E, Expression of PKD1 in tg(fli1:EGFP) zebrafish endothelial cells at 24 hpf as shown by RT-PCR of EGFP purified endothelial cells. Scale bars: 25 µm. (JPG) [file pone.0068033.s002.jpg]

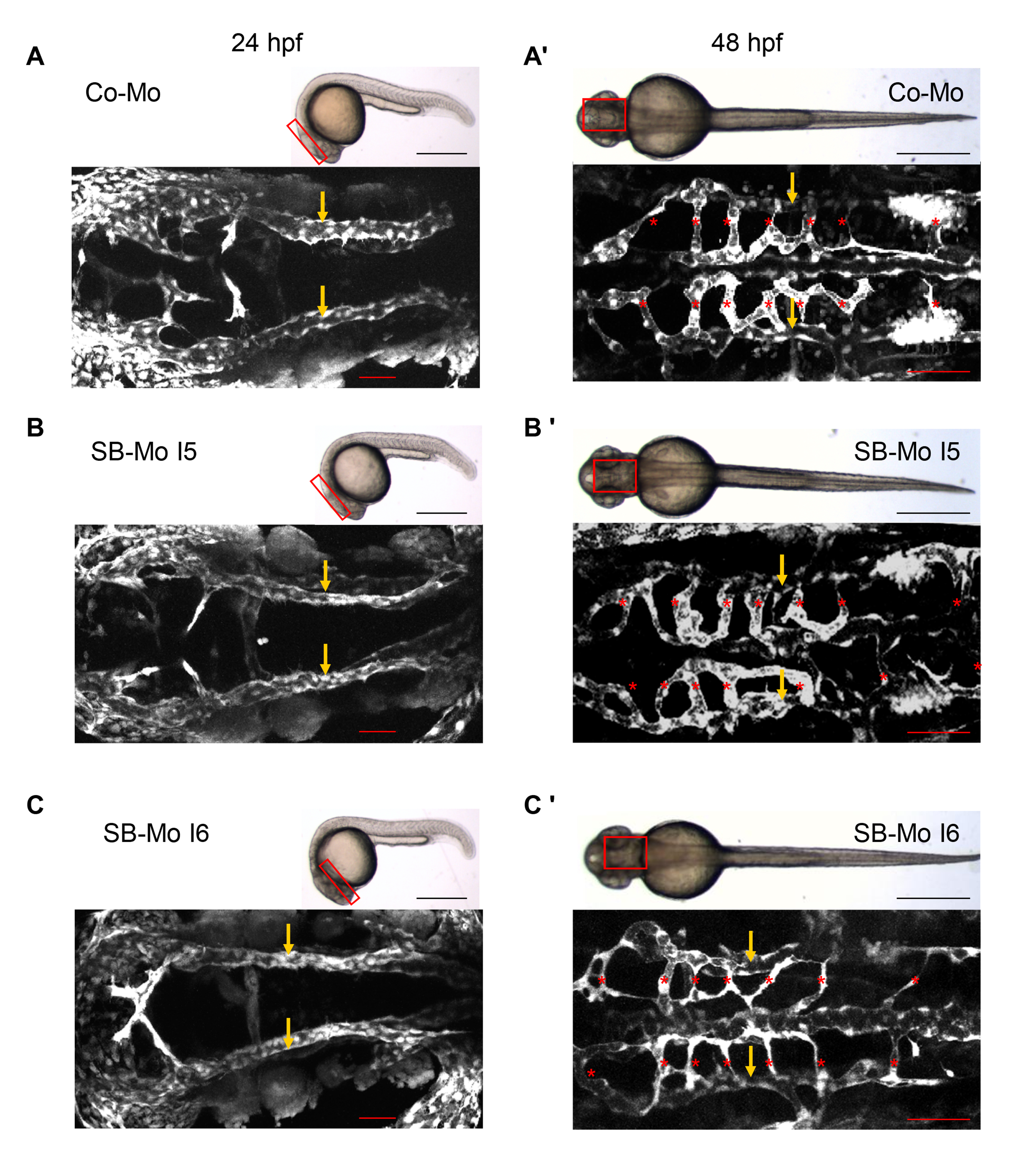

Supplement: Figure S3 — PKD1 silencing in zebrafish did not alter cranial angiogenesis. A–C′, 24 hpf (A, B, C) and 48 hpf (A′, B′, C′) tg(fli1:EGFP) zebrafish embryos were analyzed for defects in the primordial hindbrain channel (PHBC, arrows) and central arteries (CA, asterisks) by confocal microscopy. Injection of 500 pg SB-Mo I5 (B, B′) or 500 pg SB-Mo I6 (C, C′) did not reveal vascular defects in the cranial vasculature as compared to 2 ng Co-Mo injected tg(fli1:EGFP) zebrafish embryos (A, A′). Black scale bars: 500 µm, red scale bars: 50 µm (JPG) [file pone.0068033.s003.jpg]

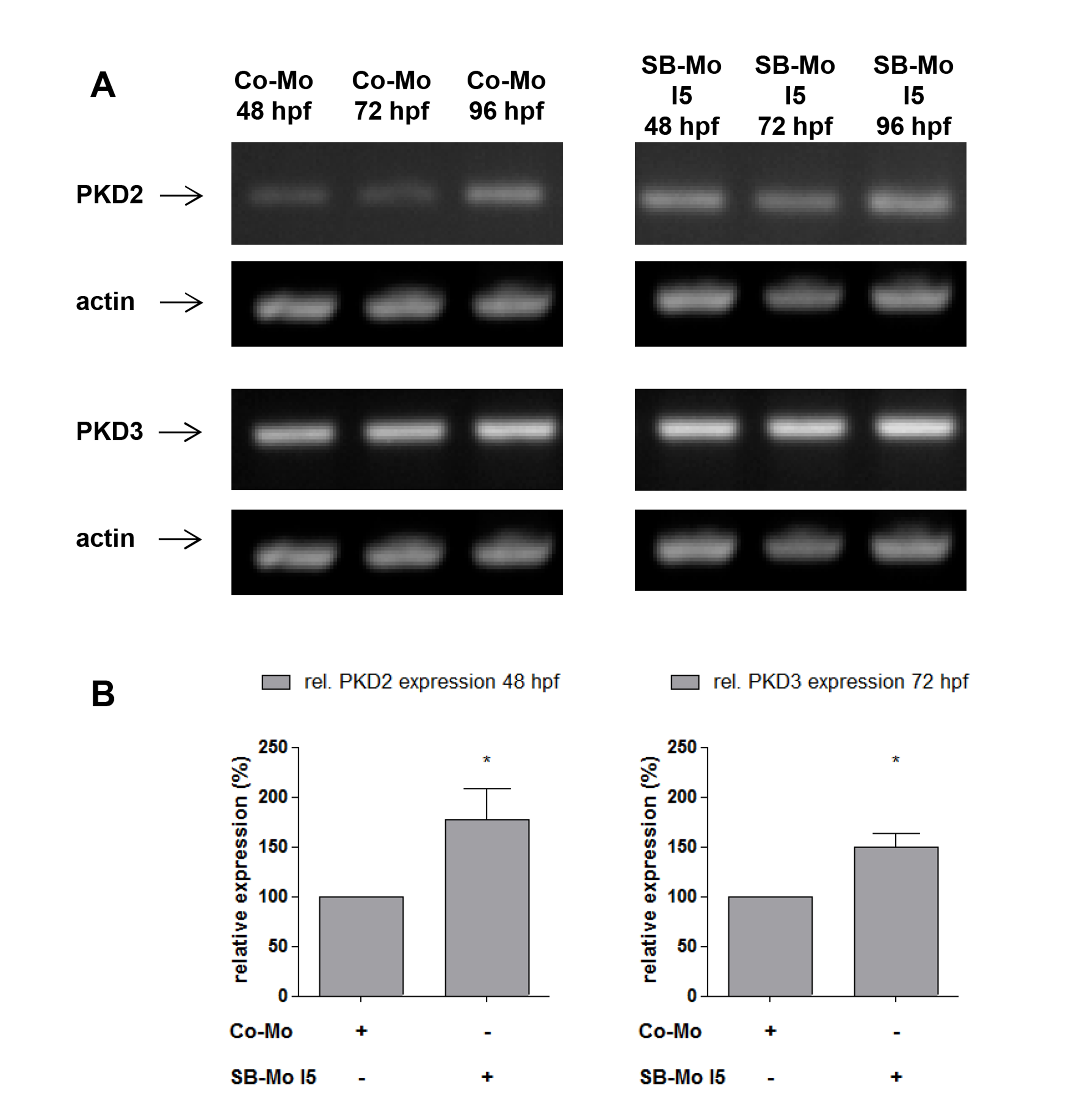

Supplement: Figure S4 — Expression of PKD2 and PKD3 in PKD1 morphants. A, RT-PCR expression analysis for PKD2 and PKD3 after PKD1 silencing in zebrafish using SB-Mo I5. Injection of SB-Mo I5 (500 pg) led to a weak increase of PKD2 and PKD3 expression at 48 hpf, 72 hpf and 96 hpf. B, Quantification of selected timepoints of (A) by densitometry; data represent means of three independent experiments with S.D. *P<0.05, **P<0.01, ***P<0.001. (JPG) [file pone.0068033.s004.jpg]

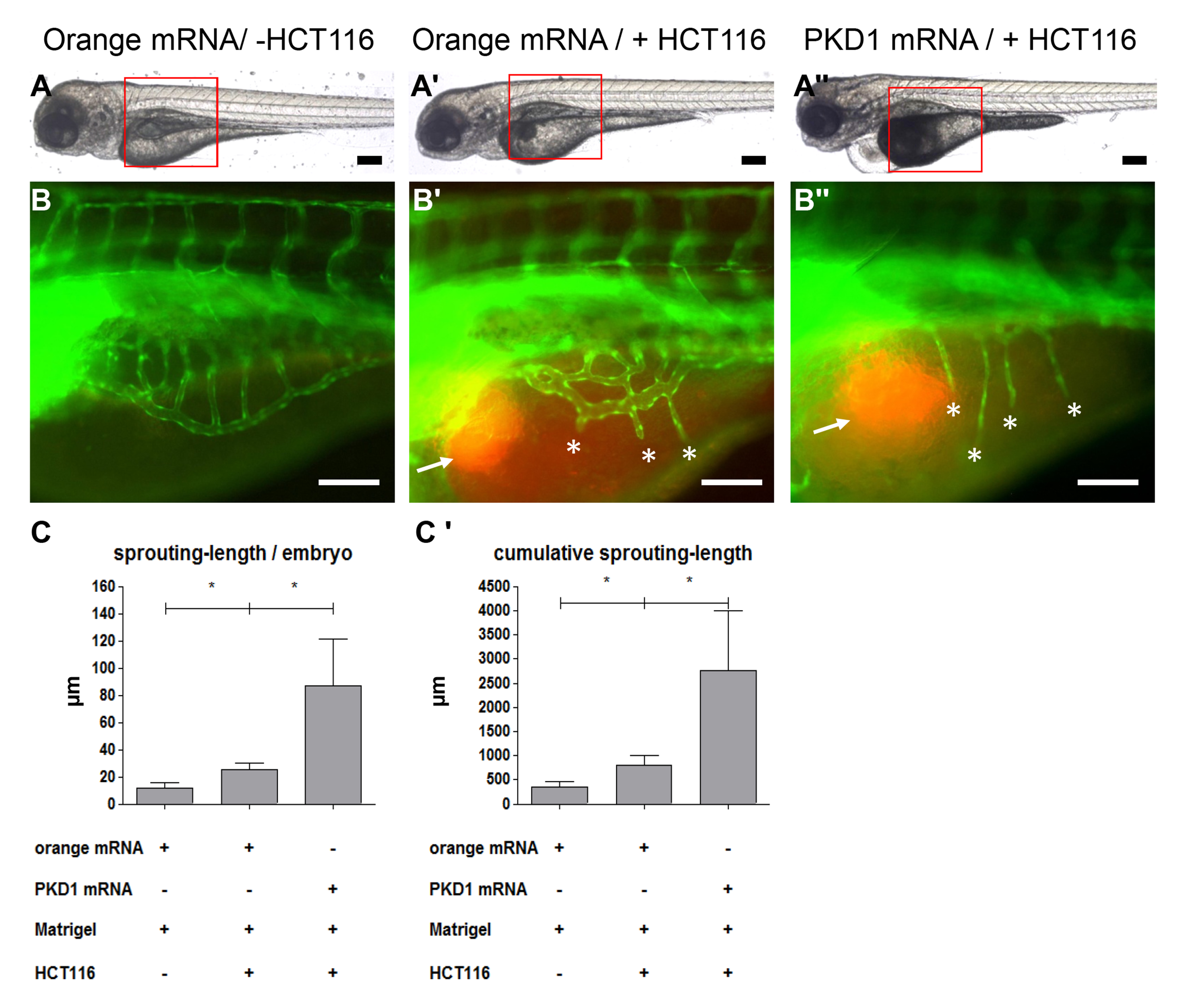

Supplement: Figure S5 — Increased tumor angiogenesis in tg(fli1:EGFP) zebrafish embryos overexpressing PKD1. Overall morphology of orange-mRNA - injected (100 pg) or PKD1 mRNA - injected (100 pg) 96 hpf embryos. At 48 hpf 1–4 nl of Matrigel (A, B) or Matrigel/HCT116 solution (A′, A″, B′, B″) was injected in the perivitelline space. Red boxes indicate regions of pictures shown in (B–B″). B–B″, HCT116 induced tumor angiogenesis as indicated by sprouting of subintestinal venous plexus (SIV) was analyzed at 96 hpf in tg(fli1:EGFP) embryos. Injection of HCT116 tumor cells (labeled with VybrantDil in red, arrow) led to a strong formation of ectopic blood vessels originated from the SIV (asterisks). In PKD1 overexpressing embryos ectopic blood vessel formation was further enhanced. C–C′, Quantification of sprouting length per embryo (C) and cumulative sprouting length (C′) with S.D. of at least 30 embryos per group. For cumulative sprouting-length all sprouts of the same number of embryos per group were summed, (C′) represents means of three independent experiments with S.D. Black scale bars: 300 µm; white scale bars: 100 µm. *P<0.05, **P<0.01, ***P<0.001. (JPG) [file pone.0068033.s005.jpg]

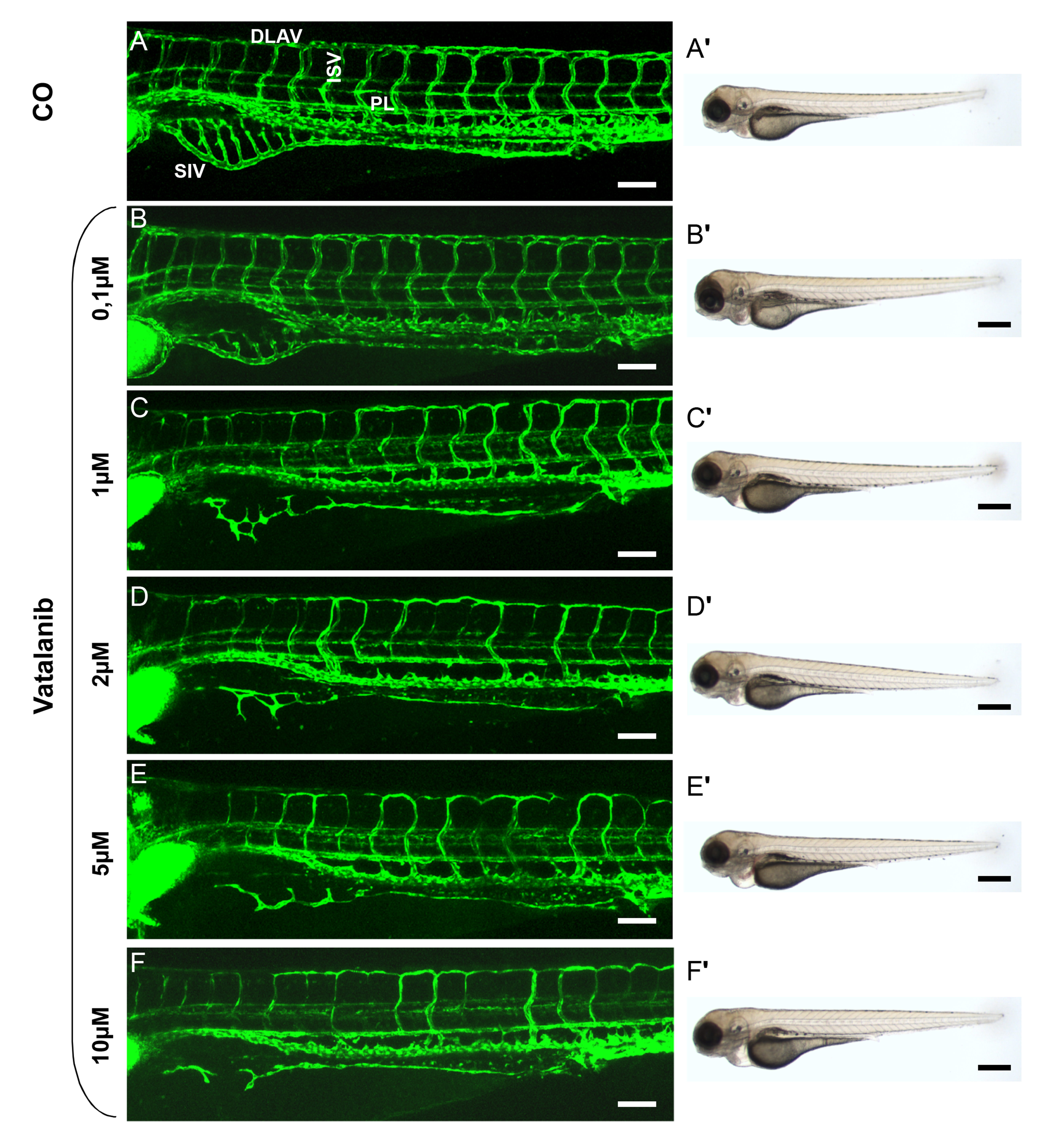

Supplement: Figure S6 — Effect of Vatalanib treatment on vascular development of the trunk vasculature in zebrafish embryos and overall morphology. A–F′, tg(fli1:EGFP) zebrafish embryos were treated with Vatalanib in different concentrations for 48 hours, beginning at 48 hpf. Confocal images of trunk vasculature (A–F) and light images of overall morphology (A′–F′) were taken at 96 hpf. Embryos were incubated with control solution (0.05% DMSO in eggwater equal to the DMSO concentration in 10 µM Vatalanib treated embryos) (A, A′), 0,1 µM Vatalanib (B, B′), 1 µM Vatalanib (C, C′), 2 µM Vatalanib (D, D′), 5 µM Vatalanib (E, E′) and 10 µM Vatalanib (F, F′). ISV = intersomitic vessel, SIV = subintestinal vein plexus, PL = parachordal lymphangioblasts, DLAV = dorsal longitudinal anastomotic vessel. White scale bar: 100 µm; black scale bar: 500 µm. (JPG) [file pone.0068033.s006.jpg]
